# Novel Animal Model of Spontaneous Cerebral Petechial Hemorrhage Using Focused Ultrasound in Rats

**DOI:** 10.3390/medicina58070881

**Published:** 2022-06-30

**Authors:** Sang-Youl Yoon, Mun Han, Chaejin Lee, Eun-Hee Lee, Moonsik Kim, Kyoung-Tae Kim, Jeong-Hyun Hwang, Sungdae Na, Juyoung Park, Ki-Su Park

**Affiliations:** 1 Department of Neurosurgery, School of Medicine, Kyungpook National University Chilgok Hospital, Kyungpook National University, Daegu 41404, Korea; customplus@naver.com; 2 Medical Device Development Center, Daegu-Gyeongbuk Medical Innovation Foundation, Daegu 41061, Korea; munhan@dgmif.re.kr (M.H.); ehlee@dgmif.re.kr (E.-H.L.); 3 Department of Neurosurgery, School of Medicine, Kyungpook National University Hospital, Kyungpook National University, Daegu 41944, Korea; cjleee01@gmail.com (C.L.); nskimkt7@knu.ac.kr (K.-T.K.); jhwang1027@gmail.com (J.-H.H.); 4 Department of Pathology, Kyungpook National University Chilgok Hospital, Kyungpook National University School of Medicine, Daegu 41404, Korea; teiroa83@gmail.com; 5 Department of Biomedical Engineering, Kyungpook National University Hospital, Daegu 41944, Korea; bluepoison14@gmail.com; 6 Department of High-Tech Medical Device, Gachon University, Seongnam 13120, Korea

**Keywords:** focused ultrasound, blood–brain barrier, acoustic cavitation, ultrasound field stimulation

## Abstract

*Background and Objectives:* Petechial cerebral hemorrhages can be caused by various factors, such as traumas, cerebral infarctions, and aging, and is related to the disruption of the blood–brain barrier or the cellular damage of blood vessels. However, there is no animal model that recapitulates cerebral petechial hemorrhages. *Materials and Methods:* Here, we implemented a petechial hemorrhage using a novel technology, i.e., microbubble-assisted focused ultrasound (MB + FUS). *Results:* This method increases the permeability of the blood–brain barrier by directly applying mechanical force to the vascular endothelial cells through cavitation of the microbubbles. Microbubble-enhanced cavitation has the advantage of controlling the degree and location of petechial hemorrhages. *Conclusions:* We thus generated a preclinical rat model using noninvasive focal MB + FUS. This method is histologically similar to actual petechial hemorrhages of the brain and allows the achievement of a physiologically resembling petechial hemorrhage. In the future, this method shall be considered as a useful animal model for studying the pathophysiology and treatment of petechial cerebral hemorrhages.

## 1. Introduction

A petechial hemorrhage occurs as a result of pericapillary bleeding in a wide range of disorders, including a traumatic brain injury (TBI), a cerebral infarction, and cerebral microbleeding, in the elderly [1]. Since their potential significance was first recognized by Tardieu in 1855, much attention has been paid to their diagnostic relevance since then [2]. A petechial hemorrhage, as the name suggests, usually appears as small petechial areas of bleeding, which are observed as an increased attenuation of the affected area on computed tomography (CT) or a loss of signal on magnetic resonance imaging (MRI) [1]. Although the pathogenesis of a petechial hemorrhage is often speculative despite its typical appearance, various pathogenesis mechanisms may be involved. These range from the mechanical disruption of capillary walls to subtle cellular damage, consequently allowing the passage of red blood cells through the endothelial cytoplasm and the blood–brain barrier (BBB) [2].

Animal models of cerebral hemorrhage can help us understand its pathogenesis and explore prophylactic or therapeutic approaches. Brain hemorrhage models have been studied in several species, including mice, rats, rabbits, cats, pigs, and primates [3]. A cerebral hemorrhage model should be carefully selected according to the purpose of the study. However, most animal models have implemented intracerebral hemorrhages, in which a large amount of hematoma is caused by the rupture of blood vessels. This hemorrhage is essentially different from a petechial hemorrhage. In addition, petechial hemorrhages have been reported as part of the accompanying bleeding in cerebral infarction and head trauma models in animal studies that were not focused on petechial hemorrhages [4,5,6,7]. Therefore, animal models that can faithfully implement and recapitulate petechial hemorrhages for its study are needed.

Magnetic resonance-guided focused ultrasound (MRgFUS) is a novel procedure that is currently being applied in a wide range of clinical fields including the neurosurgery field [8,9]. Furthermore, microbubble-assisted focused ultrasound (MB + FUS) is an advantageous, noninvasive approach for penetrating the BBB because of its modifiable and transient effects on the vasculature and its ability to target specific brain regions [10]. In particular, the main principle of MB + FUS is the acoustic response of microbubbles, which are lipid-or protein-encapsulated gas spheres of 1–10 μm in diameter. Due to their high compressibility and cavitation tendency in ultrasound, microbubbles excel at transferring the kinetic energy of traveling sound waves into the local microenvironment. Upon cavitation, the microbubbles vibrate in volume and induce fluid flow within a range of diameters at the surface. Consequently, these oscillations lead to mechanical fluid impingement and the temporary penetration of surrounding cells and tissues, due to rupture of blood vessels [10]. Therefore, we hypothesized that using a focused ultrasound, a new cerebral petechial hemorrhage model could be generated to overcome the limitations of existing models.

Herein, we propose a new experimental model of petechial hemorrhage that occurs due to trigger of cerebral microvascular injury using MB + FUS in rats. Furthermore, we performed pathological examinations and behavioral assessments to reach a better understanding of cerebral petechial hemorrhage in preclinical studies performed in a novel experimental model.

## 2. Materials and Methods

### 2.1. Study Subjects and Institutional Review Board Statement

All experiments were conducted following the procedures approved by the Daegu-Gyeongbuk Medical Innovation Foundation (DGMIF) Institutional Animal Care and Use Committee (IACUC, DGMIF-20122902-01). All procedures and animal handling were performed according to the ethical guidelines for animal research, with no pain nor animal suffering. Animals were acclimated for seven days on arrival and housed in cages at 26 °C with a 12 h light/dark cycle. Animals (12 male Sprague-Dawley (SD) rats; 9–10 weeks old; weighing 325 ± 20 g, Koatech, Pyeongtaek, Korea) were anesthetized with a mixture of zoletil (25 mg/kg) and rompun (4.6 mg/kg) before each experiment [11]. The rats were randomly divided into the following four experimental groups according to sonication intensity: (1) sham, (2) 1.5 MPa, (3) 1.8 MPa, and (4) 2.0 MPa. Three rats from each group were used in this experiment.

Interventionary studies involving animals or humans, and other studies that require ethical approval, must list the authority that provided approval and the corresponding ethical approval code.

### 2.2. Focused Ultrasound Sonication

**Figure 1:**
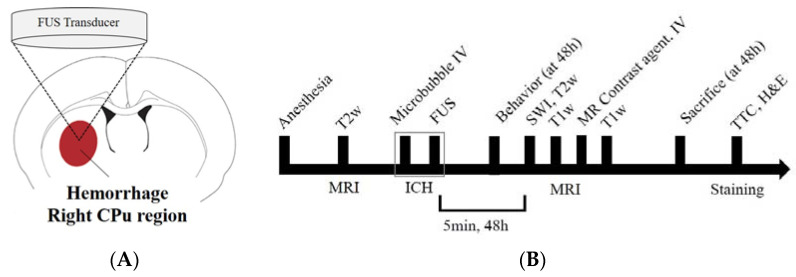
Experimental setup for focused ultrasound (FUS)-induced petechial hemorrhage and animal experimental design. (**A**) The intracerebral hemorrhage was targeted in the right caudate putamen (CPu) region for all FUS procedures. (**B**) Experimental design for investigating the FUS-induced ICH model. T2-weighted magnetic resonance (MR) images were obtained to locate the focal region before the sonication, and susceptibility weighted imaging, T2 and T1-weighted images and T1-contrast enhanced images were acquired for each time (5 min, 48 h) after the FUS. SD-rats were sacrificed and perfused for staining after 48 h after the FUS.

Rats anesthetized with Zoletil (25 mg/kg) and Rompun (4.6 mg/kg) mixture were placed on an MR-compatible animal bed in a supine position. The MRgFUS system (RK-100, FUS Instruments, Toronto, ON, Canada) was used to sonicate the rat brains for inducing the hemorrhage model. Ultrasound was generated using a single-element therapeutic FUS transducer (diameter, 75 mm; radius, 60 mm; center frequency, 1.1 MHz). The target region for sonication was the right caudate putamen (CPu). A schematic representation of the system is shown in Figure 1A. Sonication was applied synchronously to an intravenous injection of microbubbles (Definity, Lantheus Medical Imaging, N.Billerica, MA, USA), which consisted of C_3_D_8_ gas encapsulated by an outer phospholipid shell. The microbubble solution was diluted 50-fold in saline and injected intravenously at a dose of 10 μL/kg. Microbubbles with less than two hours of activation were used (manufacturer’s guidelines  <12 h) to ensure a similar level of microbubble concentration among all experiments [12]. The sonication intensities were (1) sham, (2) 1.5 MPa, (3) 1.8 MPa, and (4) 2.0 MPa.

### 2.3. Magnetic Resonance Imaging

**Figure 2:**
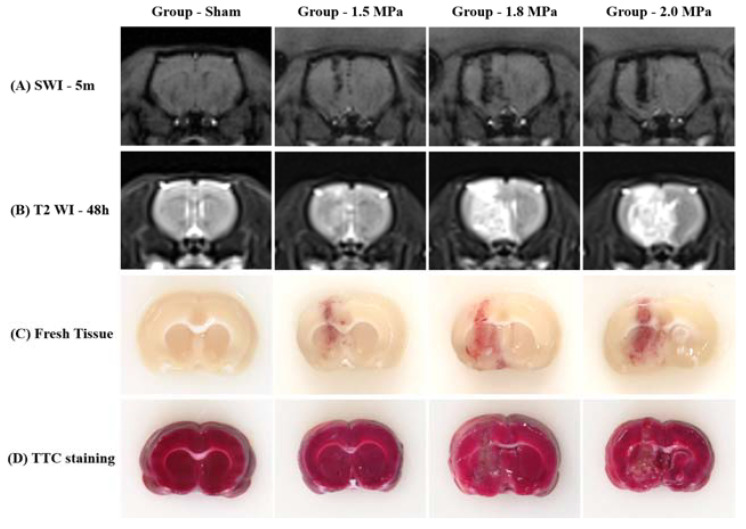
Representative MR images, fresh tissue and triphenyltetrazolium chloride (TTC) staining (**A**) SWI, (**B**) T2 WI, (**C**) Fresh tissue slice, and (**D**) TTC staining.

MRI data were acquired using a 3.0T MRI scanner (MAGNETOM Skyra; Siemens Medical Solutions, Erlangen, Germany) and a 7-cm loop coil. T2-weighted imaging was used to select sonication targets prior to FUS and evaluate perilesional edema after FUS. T1-weighted contrast-enhanced imaging was used to evaluate brain parenchymal damage or BBB-disrupted areas. Susceptibility weighted imaging (SWI) was used to assess the volume of the petechial hemorrhagic lesion. All MR images were obtained at 5 min and 48 h after FUS (Figure 1B). The following MRI parameters were employed for 2D fast spin echo T1-weighted images: field of view = 40 mm × 40 mm, matrix size = 128 × 128, axial slices = 8, slice thickness = 1.5 mm, slice gap = 0, repetition time (TR) = 500 ms, echo time (TE) = 6.5 ms, number of averages = 30, scan time = 5 min; T2-weighted images: TR = 2000 ms, TE = 33 ms, number of averages = 32, scan time = 5 min, and the other parameters were equal to those of the T1-weighted images. The SWI parameters were as follows: field of view, 50 × 50 mm; matrix size, 128 × 128; axial slices = 16, slice thickness = 1.5 mm, slice gap = 0, flip angle = 30, TR = 27 ms; TE, 20 ms; number of averages = 15, scan time, 10 min. During MRI scans, the animal’s body temperature was maintained at 35–37 °C using a warm water blanket [12]. T2-weighted MR images were obtained to locate the focal region before sonication, and SWI, T2, T1-weighted images, and T1-contrast enhanced images were acquired at each time point (5 min, 48 h) after the FUS (Figure 2).

### 2.4. Behavioral Assessment

**Figure 3:**
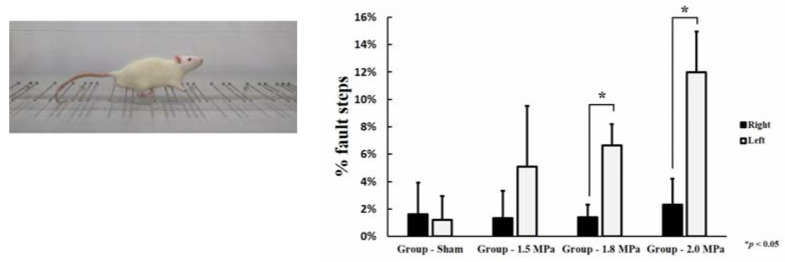
Horizontal ladder rung walking test. Statistical significance: * *p* ≤ 0.05 significant.

All behavioral assessments were performed 48 h after FUS treatment. The behavioral assessment was a horizontal ladder rung walking test [13]. The horizontal ladder rung walking test device consists of sidewalls made of clear acrylic sheet and metal rungs (3 mm diameter), which can be inserted to create a floor with a distance of at least 1 cm between rungs. The sidewalls were 1 m long and 19 cm high, as measured from the height of the rungs. The ladder was elevated 30 cm above the ground with a neutral start cage and refuge (home cage) at the end. The width of the alley was adjusted to the size of the animal so that it was approximately 1 cm wider, to prevent the animal from turning around (Figure 3). During pre-training, the animals crossed the horizontal ladder twice. After familiarization, they were recorded with a video camera. Fault steps were defined as complete misses, which represent the total number of paw misplacements per trial. All animals were randomly divided into treatment groups and evaluations were performed by two blinded observers. The recording time per subject was approximately 5 min. Video recordings were analyzed using frame-by-frame analysis.

### 2.5. Histology

For hematoxylin and eosin (H&E) staining, SD rats were sacrificed at 48 h after FUS sonication. The harvested brains were embedded in paraffin blocks and serially sectioned at 5 μm in the axial plane. H&E staining was performed using a H&E staining kit (VECTOR Laboratories, Burlingame, CA, USA). Images were acquired using a Zeiss Axio scanner. Z1 Digital Slide Scanner (Carl Zeiss, Oberkochen, Germany) and red blood cell areas in the sonicated brain region were observed using ZEN software (version 3.1; Carl Zeiss, Oberkochen, Germany). For evaluation of infarcted areas, brain sections were stained with 2% 2,3,5-tetraphenyltetrazolium chloride (TTC, T8877-25G, Sigma-Aldrich, St. Louis, MO, USA) in PBS at 37 °C for 30 min. The solution was prepared immediately prior to use and protected from light [14].

### 2.6. Statistics

Statistical analysis was performed using a commercial software (IBM Statistical Package for the Social Sciences 21.0, IBM Corp., Armonk, NY, USA). The volume of petechial hemorrhage lesions is presented as the mean ± standard deviation. The sham-control and sonication groups (group-1.5 MPa, group-1.8 MPa and group-2.0 MPa, respectively) were compared via the Kruskal–Wallis one-way analysis of variance (ANOVA) on ranks with post-hoc Tukey’s HSD test in terms of the volume of petechial hemorrhage lesion in the SWI. The comparison between the right and left fault steps in the behavioral assessment was performed using a nonparametric t-test. Differences were considered statistically significant at *p* < 0.05.

## 3. Results

### 3.1. MRgFUS System Induces Petechial Cerebral Hemorrhage with Perilesional Edema

Figure 2 shows that diffuse petechial cerebral hemorrhages occurred at the intended site in all rats according to the ultrasound intensity. According to the SWI at 5 min after sonication, MB + FUS produced hemorrhages along the tract of sonication, and a stronger intensity of FUS induced a larger amount of hemorrhage. In addition, delayed T2-WI MR images 48 h after FUS revealed diffuse perilesional edema, and the stronger intensity of FUS induced more severe perilesional edema. Fresh tissue slices also confirmed petechial hemorrhage along the tract of sonication. However, TTC staining revealed no cerebral infarctions.

### 3.2. Petechial Hemorrhage Lesion Volume

**Figure 4:**
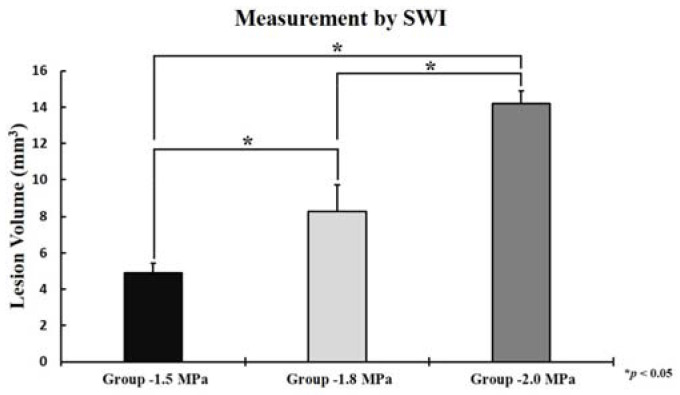
The volume of petechial hemorrhage measured by susceptibility weighted imaging(SWI), 1.5 MPa: 4.91 ± 0.53 mm^3^, 1.8 Mpa: 8.28 ± 1.43 mm^3^, and 2.0 MPa: 14.21 ± 0.70 mm^3^. The data represented were the mean ± SD (*n* = 3). Statistical significance: * *p* ≤ 0.05 significant.

Based on the SWI image, volume quantifications of petechial hemorrhage lesions were performed according to each FUS intensity (Figure 4). The sonication intensities were (1) sham, (2) 1.5 MPa, (3) 1.8 MPa, and (4) 2.0 MPa. As the FUS intensity increased, the volume of petechial hemorrhage increased, respectively (1.5 MPa: 4.91 ± 0.53 mm^3^, 1.8 MPa: 8.28 ± 1.43 mm^3^, and 2.0 MPa: 14.21 ± 0.70 mm^3^). Statistical analysis showed significant differences among three groups (1.5 MPa versus 1.8 MPa: *p* = 0.013, 1.8 MPa versus 2.0 MPa: *p* = 0.010, and 1.5 MPa versus 2.0 MPa: *p* < 0.001).

### 3.3. Behavioral Assessment

In the horizontal ladder rung walking test, all rats treated with MB + FUS were paralyzed due to a petechial hemorrhage induced in the right CPu, and more errors were made by the corresponding extremities compared to the healthy ones. As the FUS intensity increased, the error rate increased in the right extremity, respectively (1.5 MPa: 5.09 ± 0.02%, 1.8 MPa: 6.64 ± 0.016%, and 2.0 MPa: 11.97 ± 0.03%). In particular, a statistically significant paralysis was observed in the 1.8 MPa (*p* = 0.002) and the 2.0 MPa group (*p* = 0.003) (Figure 3).

### 3.4. Histology

**Figure 5:**
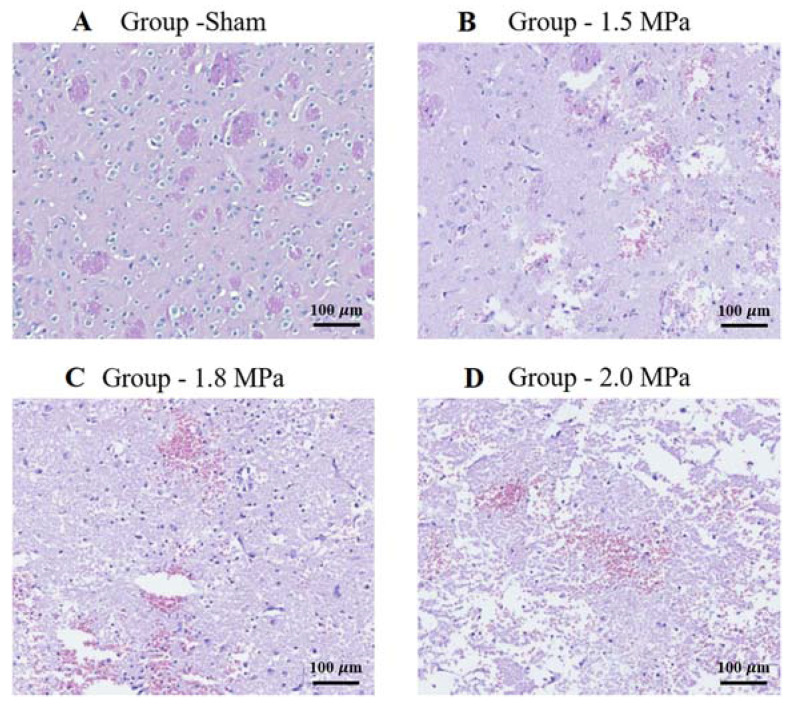
H&E staining: representative brain sections of sham and sonication. Histopathological findings in the petechial hemorrhage model using focused ultrasound in rats. As the intensity of ultrasound increases, the brain tissue shows an increased amount of gliosis, extravasated red blood cells, congestion, and destruction of small blood vessels, which is consistent with petechial hemorrhage. (**A**) Sonication intensity group sham. (**B**) Sonication intensity group 1.5 Mpa. (**C**) Sonication intensity group 1.8 Mpa. (**D**) Sonication intensity group 2.0 Mpa. Original magnification: (**A**–**D**), (150× magnification). Scalebar = 100 μm.

The histological findings in the rat petechial hemorrhage model were examined. At a low-intensity ultrasound, the brain tissue showed a mild gliosis with extravasated red blood cells compared to the control group. As the intensity increased, the brain tissue showed an increased gliosis, more extravasated red blood cells, and the destruction of small blood vessels, consistent with a petechial hemorrhage. (Figure 5).

## 4. Discussion

To date, the animal experimental models for spontaneous cerebral hemorrhages in the literature are as follows: (1) a microballoon insertion model, (2) an autologous whole blood injection model, (3) a collagenase animal model of intracerebral hemorrhage, (4) a thrombin model of intracerebral hemorrhage, and (5) a hypertensive stroke model [6]. Implementing microbleeding in animal models is challenging; one particular difficulty is to control the degree and location of the bleeding. In this sense, our ultrasound-based petechial hemorrhage model has the advantage of controlling the implementation, location, and intensity of microbleeding. Therefore, it can be used for research involving brain injuries that cause petechial hemorrhages.

**Figure 6:**
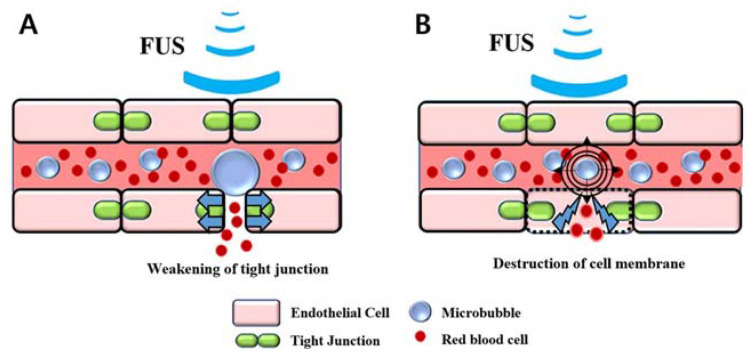
Illustration of the petechial hemorrhage caused by microbubble-assisted focused ultrasound (MB + FUS): (**A**) Blood–brain barrier disruption, (**B**) Destruction of cell membrane by microbubble-enhanced cavitation.

Our animal model has some similarities to petechial hemorrhages caused by trauma and the post-ischemic reperfusion of cerebral infarctions. On one hand, the petechial hemorrhage caused by trauma is a molecular event that occurs after an impact. The mechanical force of the impact induces the nuclear translocation of Sp1 and NF-κB, two mechanosensitive transcription factors, which in turn increase Sur1 transcriptional expression in endothelial cells. Sur1 causes oncotic (necrotic) death of endothelial cells and eventually causes physiological disruption of the capillaries, leading to capillary fragmentation [15,16,17,18,19]. This results in the extravasation of blood and a petechial hemorrhage. On the other hand, a cerebral infarction increases the permeability of tight junctions between endothelial cells. After ischemia, reperfusion occurs concomitantly with the impaired autoregulation of cerebral blood vessels, resulting in a rapid increase in cerebral blood flow at the lesion site along with an increase in the permeability of the BBB [20]. In our animal model, petechial hemorrhages were achieved by applying direct mechanical forces to the vascular endothelial cells through microbubbles and by increasing the permeability of the BBB due to microbubble-enhanced cavitation (Figure 6), which can control the degree and location of the petechial hemorrhage.

This animal model is expected to be utilized in the following areas. It can be used as a research model for the treatment and management of patients with a spontaneous cerebral hemorrhage by reproducing a mechanism similar to hemorrhagic transformation that can occur due to an increase in the permeability of the BBB after a cerebral infarction. In addition, since it can induce hemorrhage in a specific area of the brain, it will be applicable to the study of the function of that area, and this animal model may be applicable to the study of the appropriate rehabilitation treatment for insults to that area.

In this FUS-based cerebral hemorrhage animal model, the possibility of developing other hemorrhagic animal models such as a lobar hemorrhage can be glimpsed by utilizing the change in sonication intensity and microbubble application.

Our rat model of petechial hemorrhages caused by MB + FUS has several limitations. First, the pathophysiology of a cerebral hemorrhage due to TBI and spontaneous intracerebral hemorrhage is complex to analyze. In particular, secondary damage after the first impact of hemorrhage involves multiple “toxic” factors present in activated blood components, such as the infiltration of the brain by systemic immune cells, the activation of microglia, and the hematoma-induced apoptotic death of neuronal and glial cells in the surrounding parenchymal rim, followed by the progressive rupture of the blood–brain barrier and a growing brain edema [21]. Our study only showed the feasibility of implementing petechial hemorrhages and did not study the impact of secondary damage. In addition, it is unknown whether ultrasound exerts a synergistic effect with petechial hemorrhages in the brain microenvironment, thus, for example, triggering cellular apoptosis, necrosis, and immune reactions. Finally, an accurate stereotactic petechial hemorrhage could not be performed because of the insufficient number of transducers. Therefore, further studies on brain anomalies after exposure to MB + FUS are needed to apply this model for testing novel therapeutics.

## 5. Conclusions

We have shown a preclinical rat model of petechial hemorrhage using noninvasive focal MB+ FUS. This method mimics the main histological features of a petechial hemorrhage, thus allowing to achieve a highly relevant pathological state for further studies. Moreover, by adjusting some parameters and focus, the extent and location of petechial hemorrhages after trauma and infarction can be modified in a highly reproducible manner.

## Data Availability

The datasets generated during and/or analyzed during the current study are available from the corresponding author on reasonable request.
